# Herbivore-induced maize leaf volatiles affect attraction and feeding behavior of *Spodoptera littoralis* caterpillars

**DOI:** 10.3389/fpls.2013.00209

**Published:** 2013-06-28

**Authors:** Georg E. von Mérey, Nathalie Veyrat, Marco D'Alessandro, Ted C. J. Turlings

**Affiliations:** Laboratory for Fundamental and Applied Research in Chemical Ecology, Institute of Biology, University of NeuchâtelNeuchâtel, Switzerland

**Keywords:** *Spodoptera littoralis*, green leaf volatiles, maize, larval foraging behavior, host plant suitability

## Abstract

Plants under herbivore attack emit volatile organic compounds (VOCs) that can serve as foraging cues for natural enemies. Adult females of Lepidoptera, when foraging for host plants to deposit eggs, are commonly repelled by herbivore-induced VOCs, probably to avoid competition and natural enemies. Their larval stages, on the other hand, have been shown to be attracted to inducible VOCs. We speculate that this contradicting behavior of lepidopteran larvae is due to a need to quickly find a new suitable host plant if they have fallen to the ground. However, once they are on a plant they might avoid the sites with fresh damage to limit competition and risk of cannibalism by conspecifics, as well as exposure to natural enemies. To test this we studied the effect of herbivore-induced VOCs on the attraction of larvae of the moth *Spodoptera littoralis* and on their feeding behavior. The experiments further considered the importance of previous feeding experience on the responses of the larvae. It was confirmed that herbivore-induced VOCs emitted by maize plants are attractive to the larvae, but exposure to the volatiles decreased the growth rate of caterpillars at early developmental stages. Larvae that had fed on maize previously were more attracted by VOCs of induced maize than larvae that had fed on artificial diet. At relatively high concentrations synthetic green leaf volatiles, indicative of fresh damage, also negatively affected the growth rate of caterpillars, but not at low concentrations. In all cases, feeding by the later stages of the larvae was not affected by the VOCs. The results are discussed in the context of larval foraging behavior under natural conditions, where there may be a trade-off between using available host plant signals and avoiding competitors and natural enemies.

## Introduction

Maize plants attacked by herbivorous insects emit volatile organic compounds (VOCs) that attract natural enemies of herbivores (Dicke et al., 1990; Turlings et al., 1990; Turlings and Wäckers, 2004; Arimura et al., 2009). In the case of maize plants, the blend of VOCs emitted by caterpillar-damaged plants is typically composed of green leaf volatiles (GLVs, C-6 aldehydes, alcohols, and their esters), nitrogenous, and aromatic compounds, as well as mono, homo and sesquiterpenes (Paré and Tumlinson, 1999; D'Alessandro and Turlings, 2006). Among the VOCs that have been identified in these blends, GLVs have received particular attention. They are emitted upon mechanical damage, immediately after feeding on the maize plant begins (Turlings et al., 1998), and have been considered important for the innate attraction of parasitoids, as they are emitted in higher amounts by freshly damaged plants than by plants with only old damage (Whitman and Eller, 1990; Hoballah and Turlings, 2005). Commonly, insect herbivores are repelled by inducible plant volatiles (Bernasconi et al., 1998; De Moraes et al., 2001; Rostas and Hilker, 2002). This is particularly evident for Lepidoptera (De Moraes et al., 2001), but this is not true for all herbivores. In particular coleopterans are known to be attracted to previously infested plants (Bolter et al., 1997; Landolt et al., 1999) and they may be attracted to GLVs as was found for scarab (Hansson et al., 1999) and buprestid beetles (de Groot et al., 2008), and flea beetles (Halitschke et al., 2008).

Interestingly, larval stages of several Lepidoptera are attracted by volatiles emitted by plants that have been damaged by conspecific larvae. This was found for neonates of several Lepidoptera species, including *Ostrinia nubialis* (Hübner) and *Ostrinia furnacalis* (Guenée) (Lepidoptera: Pyralidae) on maize (Huang et al., 2009; Piesik et al., 2009), *Spodoptera frugiperda* (J. E. Smith) (Lepidoptera: Noctuidae) on maize and cowpea (Carroll et al., 2006, 2008), and *Estigmene acrea* (Drury) (Lepidoptera: Arctiidae) on soybean, tomato, and maize (Castrejon et al., 2009). Furthermore, caterpillars adapt their behavior depending on plant VOC emission (Shiojiri et al., 2006). This attraction to VOCs emitted by already infested host plants is puzzling, as it will lead to competition and may increase the risk of cannibalism and attack by natural enemies that are attracted to the same volatiles. Cannibalism is common among noctuid larvae, such as *Spodoptera littoralis* (Boisduval) (Abdel Salam and Fokhar. cited in Fox, 1975), *S. frugiperda* (Chapman et al., 1999), and *Helicoverpa armigera* (Hübner) (Kakimoto et al., 2009). The attraction of natural enemies to herbivore-induced volatiles has been shown for numerous tritrophic systems (Dicke et al., 1990; Turlings et al., 1990; Heil, 2008; Dicke and Baldwin, 2010; Hare, 2011), which makes one wonder why lepidopteran larvae are attracted to the same volatiles. This apparent maladaptive behavior may be explained by a trade-off between risks: in the field harsh weather conditions and attempts to escape parasitoids and predators cause larvae to frequently fall off plants (personal observ.). In order to find back the same plant or new suitable plants the larvae will have to rely on dependable and available VOC signals. Induced VOCs may provide the best cues, as undamaged plants are often virtually odorless (Turlings et al., 1990). However, once on a plant, caterpillars may prefer sites with minimal VOC emissions, where it is less likely to encounter competitors and natural enemies.

We therefore hypothesized that caterpillars may initially be attracted to induced VOCs, but once they are on the plant they will feed preferentially in places with low GLV emissions. We tested this for larvae of the noctuid moth *Spodoptera littoralis* (Boisduval). First we confirmed attraction to induced plant volatiles in a four-arm olfactometer and then tested their growth rate as a measure of feeding behavior when they were exposed to GLVs. Previous feeding experiences were also taken into consideration, as larval attraction may be higher for volatiles that are emitted by plant species on which the larvae previously fed (Carlsson et al., 1999).

## Materials and methods

### Plants and insects

Maize plants (*Zea mays*, cv. Delprim) were grown individually in plastic pots (10 cm high, 4 cm diameter) with commercial potting soil (Ricoter Aussaaterde, Aarberg, Switzerland) and placed in a climate chamber (23°C, 60% r.h., 16:8 h L:D, 50000 lm/m^2^). Maize plants used for the experiments were 10–12 days old and had three fully developed leaves. The evening before the experiments, plants were transferred into glass vessels, as described in Turlings et al. (2004) and kept under laboratory conditions (25 ± 2°C, 40 ± 10% r.h., 16:8 h L:D, and 8000 lm/m^2^). *S. littoralis* larvae were reared from eggs provided by Syngenta (Stein, Switzerland). The eggs were kept in an incubator at 30.0 ± 0.5°C until emergence of the larvae. Subsequently, they were transferred on artificial diet at room temperature (24 ± 4°C).

### Olfactometer experiments

Two olfactometer experiments were performed with fourth-instar *S. littoralis* larvae. In the first experiment, the attraction of larvae to an *S. littoralis*-infested maize plant vs. healthy maize plant was compared. In the second experiment, the attraction of larvae to a maize plant with fresh (mechanically inflicted) damage was tested against a plant with old (mechanically inflicted) damage. In both experiments, the effect of previous feeding experience (either artificial diet or maize) was compared. All the larvae were initially reared on artificial diet as previously described (Turlings et al., 2004). Twenty-four hours before each experiment, 90 larvae were transferred on fresh maize leaves (maize feeding experience), and 90 on artificial diet (artificial diet feeding experience).

### Attraction of fourth-instar *S. littoralis* larvae to infested maize plants

**Figure 1:**
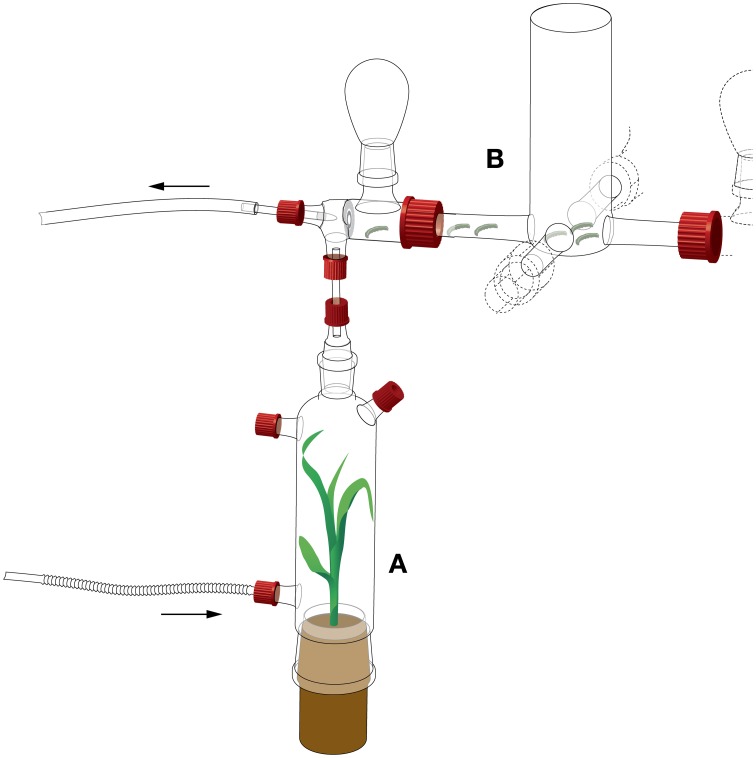
**Detail of the four-arm olfactometer setup for *S. littoralis* larval behavior. (A)** Odor source. **(B)** Choice arena. Arrows indicate airflows. Four odor sources were compared, attached to each of the four arms of the choice arena. Drawing by Thomas Degen (www.thomas-degen.ch).

A four-arm olfactometer (as described in D'Alessandro and Turlings, 2005) was modified to measure the attraction of *S. littoralis* larvae. The olfactometer consisted of a central glass choice arena (Figure 1) [6 cm internal diameter (ID), 5 cm length] with four arms (15 mm ID, 5 cm length), each with a glass elbow (5 cm length) and an upward connection for a glass bulb (50 ml). To avoid visual distraction of the larvae, a white cardboard cylinder was placed around the central choice arena.

The choice arena was connected to four glass bottles. One bottle contained a maize plant (cv Delprim) infested with 15 second-instar *S. littoralis* larvae that had been placed on the plant 16 h before the bioassay. The opposite bottle contained a healthy maize plant. The two remaining bottles remained empty. The position of the odor sources was changed between each experimental day, with the two odor sources always opposite to each other.

Thirty fourth-instar larvae were placed in a small plastic box (2 × 2 × 0.8 cm) with an open top, which was introduced in the center of the choice arena. The larvae would crawl out of the box into the central choice arena and a number of them entered one of the four arms. After 60 min, the number of larvae in each arm was counted. The larvae that did not leave the choice arena after 60 min were considered as having made “no choice” and all the larvae were removed from the olfactometer. Six such releases were done on a given day and this was repeated on 6 different days (*n* = 6).

### Attraction of fourth-instar *S. littoralis* larvae to plants with old vs. plants with fresh damage

The same setup as described above was used, with the same experimental procedure, except for the odor sources. Two maize plants were brought to the laboratory 16 h prior to the bioassay. One plant was scratched on the underside of the two oldest leaves, damaging approximately 2 cm^2^, on both sides of the central vein (Hoballah and Turlings, 2005). Caterpillar regurgitant, collected as described in Turlings et al. (1998), was applied to the two wounds. Both plants were then placed in a glass bottle and exposed to a carbon-filtered, humidified airflow of 300 ml/min for 15 h. The second plant was then scratched and regurgitant was applied. The two plants were then placed opposite to each other in the olfactometer, leaving two empty bottles between them. The airflow was then increased to 1200 ml/min through each bottle, of which 500 ml/min entered the olfactometer choice chamber. The position of the treatments was changed for each experimental day.

### GLV dispensers

To expose larvae to green leafy volatiles we made dispensers as described by von Mérey et al. (2011). The GLVs were first mixed together in an Erlenmeyer flask (100 mL) placed in ice. The composition of the mixture was 80% cis-3-hexen-1-al [92.5% purity, (NEAT), Bedoukian Research Inc., USA]; 10% cis-3-hexen-1-ol (>98%, GC, Sigma-Aldrich, CH-9471 Buchs, Switzerland); 8% cis-3-hexenyl acetate (>98%, SAFC Supply Solutions, 3050 Spruce street, St. Louis, MO 63103, USA); and 2% trans-2-hexenol (99%, ACROS Organics, New Jersey, USA). The mix was stored at -70°C until it was used. For the assays, 0.2 mL of the GLV mix was transferred into a 2 mL amber glass vial (11.6 × 32 mm) (Sigma-Aldrich, CH-9471 Buchs, Switzerland) containing clean fiberglass wool. Each vial was sealed with a PTFE/rubber septum pierced by a Drummond 2 μL micro-pipette in black polypropylene cap. This device allowed the constant release of GLVs, and their release rate was calibrated to the amount of GLVs that was found to be released by infested maize plants (*Zea mays* cv Delprim) (von Mérey et al., 2011). Control dispensers consisted of glass vials only containing fiberglass wool.

### VOC-exposure experiments

Three experiments were conducted to measure the effect of VOCs on the growth of *S. littoralis* larvae. In the first experiment, the larvae were exposed to the volatiles of caterpillar-damaged maize plants. In the second experiment, they were exposed to amounts of a blend of synthetic GLVs that fall within the range of what is commonly emitted by a single, caterpillar-infested maize plant (see von Mérey et al., 2011 for details). In the third experiment, they were exposed to high concentrations of synthetic GLVs. In all three experiments we recorded, besides weight gain, mortality, and pupation of the larvae.

### Effect of exposure to VOCs emitted by caterpillar-damaged maize plants on feeding rate of *S. littoralis* larvae

**Figure 2:**
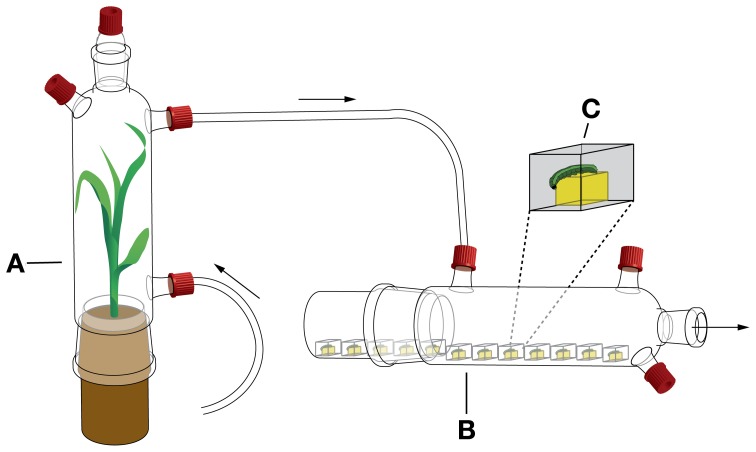
**Design of growth performance experiment. (A)** Odor source bottle, which contained either a healthy maize plant or a caterpillar-damaged maize plant. **(B)** Bottles containing 12 larvae inside small plastic boxes. **(C)** Plastic box enlarged showing a *S. littoralis* larva feeding on a cube of artificial diet. Arrows indicate the direction of the airflow.

Second-instar *S. littoralis* were placed individually inside small plastic boxes (2 × 2 × 1.5 cm) that were covered with fine-meshed nylon tissue, fixed with an elastic band. The larvae were provided a 1 cm^3^ cube of wheatgerm-based artificial diet (Turlings et al., 2004), which was changed every second day. Twelve such boxes were placed inside a glass bottle lying on its side, connected at its base with a Teflon tube to the top of an odor source bottle (Figure 2; see Turlings et al., 2004 for details on glass bottles and tubing). Odor source bottles contained either a maize plant infested with fifteen second-instar *S. littoralis* larvae (induced plant, VOCi, replaced with a new infested plant every third day) or an uninfested maize plant (control plant, VOCu, also replaced every third day). The odor source bottle was connected to a four-port air-delivery system (Model VCS-HADS-6AF6C6B; ARS Analytical Research Systems, Gainesville, FL, USA), providing a purified and humidified airflow of 300 ml/min. Two such four-port air-delivery systems were used simultaneously to introduce odors into eight exposure chambers, resulting in 48 larvae for each treatment.

Before placing the larvae inside the plastic boxes, they were weighed on a microbalance (Model MX5, Mettler Toledo, Greifensee, Switzerland). Weighing was repeated at the following time-points after placing the boxes inside the glass bottle: 5, 24, 48, 96, 144, 192, 240, 288, 312, 336, 360, 408, and 432 h. After this time-point, all larvae had pupated or had died and the experiment was terminated.

### Effect of exposure to synthetic GLVs on weight gain of *S. littoralis* larvae

The same setup as described above was used for this experiment. In this case, the odor source bottles containing a dispenser built up as follows: a 2 ml amber glass vial (11.6 × 32 mm; Sigma-Aldrich, Buchs, Switzerland) containing 100 mg clean fiberglass wool. The vial was sealed with a PTFE/rubber septum (Sigma-Aldrich, Buchs, Switzerland) pierced with a 2 μl micro-pipette (Drummond, Millan SA, Plan-Les-Ouates, Switzerland). The length of the pipette was calibrated to release a controlled amount of GLVs, similar to the amount emitted by maize plants (cv Delprim). The GLV mixture consisted of 80% (*Z*)-3-hexen-1-al [92.5% purity, (NEAT), Bedoukian Research, Danbury, CT, USA], 10% (*Z*)-3-hexenyl-acetate (<98%, SAFC Supply Solutions, St. Louis, MO, USA), 8% (*Z*)-3-hexenyl-Acetate (≥98%, SAFC Supply Solutions, 3050 Spruce Street, St. Louis, MO 63103, USA), and 2% (*E*)-2-hexenol (99%, ACROS Organics, Geel, Belgium). The same GLV dispenser was kept for the duration of the assay. Control bottles contained no dispenser.

In this experiment, the weighing of the larvae was repeated at 5, 12, 24, 48, 96, 120, and 144 h after placement in the bottles. The experiment was terminated at 144 h because the tests showed that larval weight was not affected by the volatiles at these concentrations.

### Effect of exposure to high concentrations of GLVs on weight gain of *S. littoralis* larvae

In this experiment, larvae were placed individually in a plastic box (7.5 × 6.5 × 5 cm) containing a GLV dispenser (described above), and a piece of diet (2 × 1.5 × 1 cm). The box was closed, in order to increase the concentration of GLVs. As a control, an empty dispenser was placed inside the cage without GLVs inside. There were twelve larvae in each treatment and they were weighed before placing them inside the boxes. They were weighed again after 3, 6, 9, 12, 15, 24, 40, 48, 51, 54, 58, 72, 96, 120, and 168 h.

The larger plastic boxes allowed for more mobility, compared to the cages used in the previous experiments. In order to observe whether the high concentrations of GLV affected larval mobility, we recorded whether the larvae were on the diet or off the diet during the first 8 h of exposure.

### Statistical analysis

VOC-exposure data were compared using Student's *t*-test, provided they met the assumptions of normality (Shapiro-Wilk test) and equal variance (Levene's test). Else, a Mann-Whitney test was applied. Both treatments (VOCu and VOCi exposure) were compared at each time-point individually. Data on mortality and pupation of the larvae compared using a one-way analysis of variance (ANOVA). Data was tested with SigmaStat (version 3.5, STATCON, Witzenhausen, Germany). Data on mobility were analyzed in a general linear model (GLM) with binomial distribution (the larvae were observed either on the diet or off the diet) family in R (R Development Core Team, 2009). Olfactometer data was analyzed using the software package R (R Development Core Team, 2009), in a GLM, allowing to compensate for over-dispersed data, as previously described (D'Alessandro and Turlings, 2005; Tamò et al., 2006; Ricard and Davison, 2007). This means that any positional biases or effects of the individuals on each other's behavior are considered in the model and that calculated statistical differences are solely the result of differential attractiveness of the odor sources.

## Results

### Attraction of fourth-instar *S. littoralis* larvae to induced maize plants

**Figure 3:**
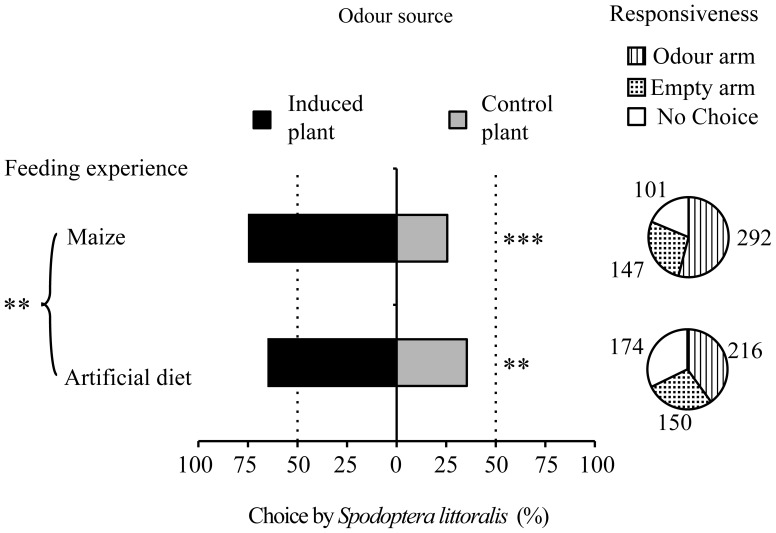
**Effect of feeding experience on the attraction of *S. littoralis* larvae to induced maize plants.** Pie charts indicate overall responsiveness (number of larvae entering the different types of arms). GLMs were performed to test for differences between arms within each group of feeding experience, as well as to compare feeding experiences. ^**^P < 0.01, ^***^P < 0.001.

The larvae that had fed on maize and the larvae fed on artificial diet were both more attracted toward caterpillar-damaged maize plants than to intact plants (GLM *P* < 0.001 and *P* < 0.002, respectively; Figure 3). However, the maize-fed larvae were attracted more strongly by the induced plants than the diet-fed larvae (GLM *P* < 0.005). Maize-fed larvae also displayed an increased responsiveness (80% entering an arm) compared to diet-fed larvae (66%).

### Attraction to old vs. fresh damage

**Figure 4:**
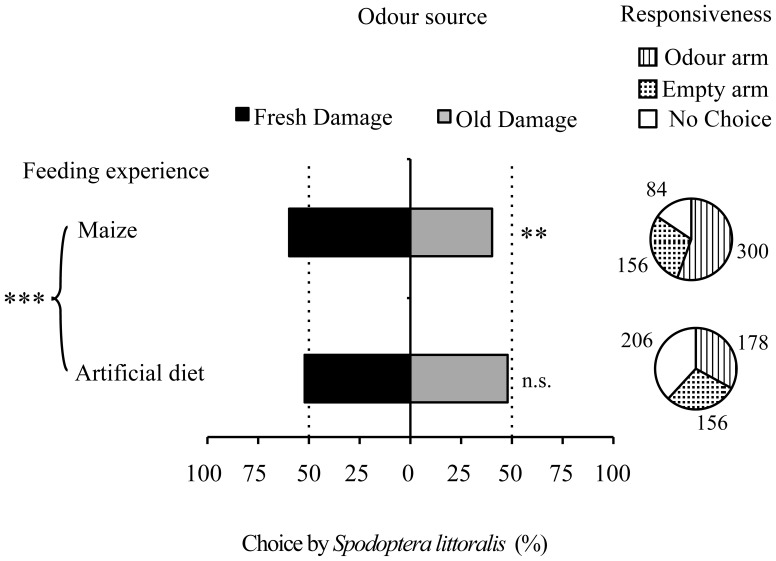
**Effect of feeding experience on the attraction of *S. littoralis* larvae to old and fresh damaged maize plants.** Pie charts indicate overall responsiveness (number of larvae entering the different types of arms). GLMs were performed to test for differences between arms within each group of feeding experience, as well as to compare feeding experiences. n.s., no significant difference (*P* > 0.05); ^**^P < 0.01, ^***^P < 0.001.

Freshly damaged plants were more attractive to maize-fed larvae (GLM *P* < 0.003) than plants with older damage (Figure 4). Artificial diet-fed larvae did not show a preference between old and fresh damage. This difference in preference between maize-fed and diet-fed larvae was significant (GLM *P* < 0.001). Also in this case, overall responsiveness of maize-fed larvae (84%) was higher than the responsiveness of artificial diet-fed larvae (62%).

### Exposure to VOCs from caterpillar-damaged maize plants

**Figure 5:**
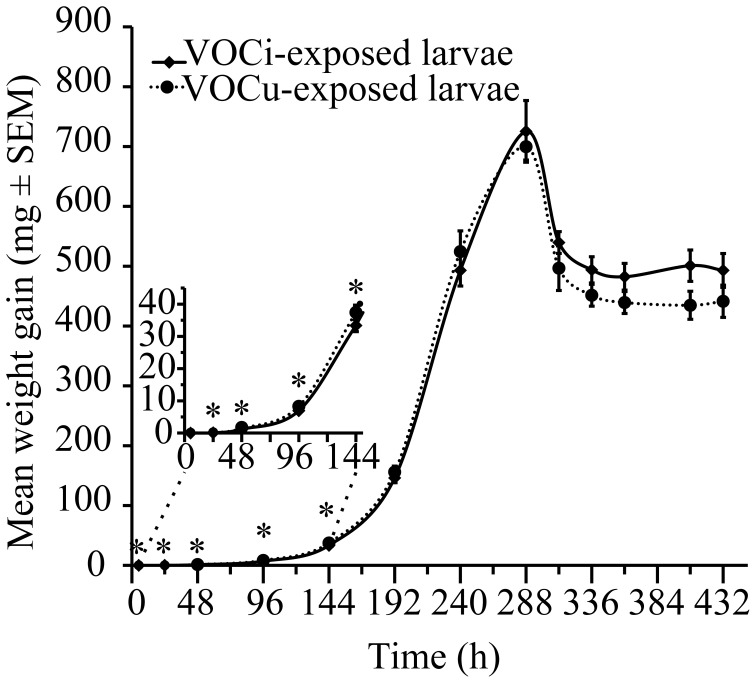
**Mean weight gain (mg ± SEM) of *S. littoralis* larvae exposed to VOCs emitted by *S. littoralis*-induced (VOCi) or healthy (VOCu) maize plants.** ^*^indicates a significant difference at *P* < 0.05 (Student's *t*-test).

The larvae that were exposed to the VOCs emitted by caterpillar-damaged maize plants grew more slowly in the early stages of development (Figure 5). Initial weight of the larvae was equal across treatments. After 5 h, there was still no difference between the two treatment groups (*P* < 0.356). However, after 24 h, the larvae exposed to VOCs from damaged plants (VOCi) had gained significantly less weight than the larvae exposed to VOCs emitted by healthy plants (VOCu) (*P* < 0.030). This difference in growth rate persisted throughout the early weighing time points: 48 h (*P* < 0.030), 96 h (*P* < 0.012), 144 h (*P* < 0.033). After this, both treatment groups displayed similar weight gains until pupation. The weight of the pupae did not differ significantly (*P* < 0.916). There was also no difference in mortality between the larvae of the two treatment groups (*P* < 0.839).

### Effect of exposure to low concentrations of synthetic GLVs on weight gain of *S. littoralis* larvae

When larvae were exposed to the synthetic volatile blend we measured no difference either in larval weight gain (5 h: *P* < 0.759, 12 h: *P* < 0.286, 24 h: *P* < 0.267, 48 h: *P* < 0.502, 72 h: *P* < 0.506, 96 h: *P* < 0.833, 120 h: *P* < 0.833, 144 h: *P* < 0.646), or mortality (0% in both treatments).

### Effect of exposure to high concentrations of GLVs on weight gain of *S. littoralis* larvae

**Figure 6:**
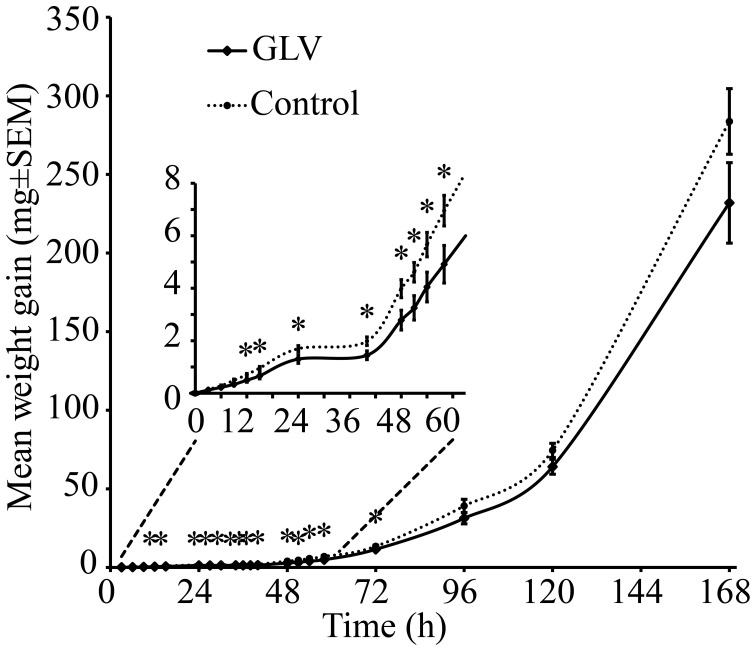
**Weight gain (mg ± SEM) of *S. littoralis* larvae exposed to GLV dispensers or control dispensers.** An asterisk above the value point indicates significant difference between treatments (*P* < 0.05 Student's *t*-test).

When larvae were exposed to high concentrations of GLVs, such as can be expected to be present in the immediacy of the feeding sites on the maize plants, the larvae were found to gain less weight at the early stages of their development (Figure 6). After 3 h (*P* < 0.514) and 6 h (*P* < 0.173), there was still no difference between the treatments. After exposure to GLVs for 9 h a strong trend of lower weight gain in GLV-exposed larvae was observed (*P* < 0.051) and at 12 h the difference between the two treatments was significant (*P* < 0.025). This difference persisted throughout the early part of the experimental time (15 h: *P* < 0.036; 24 h: *P* < 0.027; 40 h: *P* < 0.031; 48 h: *P* < 0.030; 51 h: *P* < 0.033; 54 h: *P* < 0.039; 58 h: *P* < 0.038; 72 h: *P* < 0.047). From 96 h, however, there was no longer a difference in weight gain between the treatments. Interestingly, the mobility of GLV-exposed larvae was slightly increased (*P* < 0.060), with a significant difference in number of larvae moving in the box after 6 h (*P* < 0.048). However, at 30 min (*P* < 0.410), 2 h (*P* < 0.716), 4 h (*P* < 0.572), and 8 h (*P* < 0.423), GLV-exposed and control larvae were equally on the diet and off the diet.

## Discussion

We show here that *Spodoptera littoralis* caterpillars are attracted to volatiles from maize plants that are under attack by conspecifics. This confirms the findings by Carroll et al. (2006, 2008), who obtained similar results for a related species, *S. frugiperda*, which was found to be attracted to inducible volatiles emitted from maize and cowpea seedlings. Similarly, neonate larvae of the codling moth, *Cydia pomonella*, are more attracted to apple fruits with other codling moth larvae than to uninfested fruits (Landolt et al., 2000). This is somewhat surprising, as these Lepidoptera are not known to aggregate, unlike many Coleoptera, for which both adults and larvae are often attracted to the volatiles of already infested plants (Crowe, 1995; Bolter et al., 1997; Müller and Hilker, 2000; Kalberer et al., 2001; Heil, 2004; Yoneya et al., 2010). It should be noted that in the case of *S. frugiperda*, Carroll et al. (2008) found linalool to be particularly attractive. This terpene alcohol is in fact also released, be it in lesser amounts, by undamaged maize plants, at least in some varieties (Degen et al., 2004), and therefore can be a reliable cue for the presence of maize in general. In adult Lepidoptera, however, increased linalool levels decreased oviposition (De Moraes et al., 2001; Kessler and Baldwin, 2001).

The larval response to herbivore-induced volatiles is in contrast to what is known for adult Lepidoptera, which avoid to oviposit on plants that are already under caterpillar attack (Landolt, 1993; De Moraes et al., 2001; Kessler and Baldwin, 2001; Huang et al., 2009). Such avoidance of already infested plants, which is also the case for aphids (Bernasconi et al., 1998), is expected, as it reduces the chances of competition and cannibalism, as well as predation and parasitism by natural enemies that are attracted to the same volatiles. Then why are the larvae attracted to volatiles that are indicative of these risks? To answer this it may help to list the potential disadvantages and discuss counter arguments why these may not be as important as potential advantages. The apparent disadvantages are: (1) VOCs emitting plants have mobilized their defenses and should be less suitable for caterpillar development, (2) The VOCs indicate the plants carry other larvae that will compete for the same resource and may even pose a cannibalism risk, (3) The VOCs are attractive to natural enemies of the caterpillars and therefore indicate a higher risk of predation and parasitism.

As for the counter argument, the most obvious reason to use herbivore-induced VOCs is the same as has been argued for the natural enemies (Vet and Dicke, 1992), the induced VOCs are emitted in large amounts and are therefore easily detectible and reliable cues for the presence of a host plant. Moreover, the alternative, the avoidance of inducible defenses by opting for healthy plants gives only an advantage for a very short period of time, as maize plants respond very rapidly, within hours, to an attack (Turlings et al., 1998). This is particularly true for plants that are neighboring already attacked plants and have their defenses primed in response to the volatiles emitted by the neighbor (Ton et al., 2007). This then only leaves the risk of competition and possibly cannibalism. This risk may be minor in light of the possibility of not finding a plant at all and unlike *S. frugiperda*, *S. littoralis* is not cannibalistic, at least not the colony that we used in our experiments. We therefore hypothesized that *Spodoptera* and other larvae of herbivorous insects have adapted to use the readily available and reliable herbivore-induced volatile signals to find host plants despite the risks they will face on these plants, because the likely alternative would be starvation. A similar argument formulated by Carroll et al. (2006) emphasizes the limited range at which caterpillars can forage, as compared to the highly mobile adults. The far less mobile caterpillars, when fallen to the ground, have a high risk of predation and are fully exposed to unfavorable environmental conditions. Getting back on a plant should be high priority and in most cases the same plant will be the closest to crawl on. This may also explain why we found that a previous feeding experience has a significant impact on the attractiveness of the induced maize volatiles. Similar preferences for familiar odors in *S. littoralis* larvae were found by Anderson et al. (1995) and Carlsson et al. (1999) when they studied the caterpillar's responses to cotton volatiles. This effect of experience even extends to the adult moth, which prefers to oviposit on the same plant species on which it fed as a larva (Anderson et al., 1995). It is also known that caterpillars adapt their feeding physiology to plant diet on which they feed as neonates and will perform worse on an alternative diet (el Campo et al., 2001; Zalucki et al., 2002), the more reason for the larvae to forage for the same plant species.

Once on an already infested plant, however, caterpillars could lessen the risks of competition/cannibalism, which can be very severe in certain *Spodoptera* species (Chapman et al., 1999, 2000; Richardson et al., 2009), but this is not the case for *S. littoralis*. They will also reduce the risk of predation and parasitism by avoiding the most odorous plant parts (Turlings and Wäckers, 2004). This notion is tentatively supported by the effects of maize VOCs on caterpillar feeding behavior. *S. littoralis* larvae that were exposed to the VOCs induced by their conspecifics on maize plants were found to feed and grow less than larvae that were not exposed to the VOCs (Figure 3). This is indicative of an avoidance of the VOCs, which was only evident at high concentrations. Hence, the results of the current study support our hypothesis that on a plant the caterpillars prefer to commence feeding away from freshly damaged areas, i.e., sites from which large amounts of GLVs are emitted. Yet, alternative explanations should be considered. For instance, the larvae that were exposed to GLVs volatiles might have been attracted and searched for the source of the volatiles and therefore ate less on the diet that they were offered. We can also not exclude a direct (toxic) effect of the volatiles on the larvae.

In summary, we show here that *Spodoptera littoralis* larvae are attracted to the volatiles emitted by plants that are already damaged by conspecific larvae. Although such plants are less suitable for the larvae than undamaged plants, the larvae may simply opt to go for readily detectable signals. The notion that the larvae are attracted to reliable, familiar volatile signals even if it leads them to sub-optimal resources is further supported by the fact that previous experience with the odors enhances their attractiveness. But once they are on the plants they seem to avoid the volatiles and eat less when they detect high concentrations of them. We speculate that by doing so the larvae avoid the parts of the plant with up-regulated defenses, competition/cannibalism, and natural enemies that are attracted to the same volatiles.

An understanding of signals that are of importance for host plant foraging by caterpillars can be of use in the development of pest control strategies. In this context, current focus is on foraging of adults and this has found good use in “push-pull” strategies (Khan et al., 2000, 2008; Cook et al., 2007). Similarly, with the right combination of repellent and attractive volatiles, it may be possible to manipulate the foraging of caterpillar such that they are guided away from the crop and toward their demise on trap plants.

### Conflict of interest statement

The authors declare that the research was conducted in the absence of any commercial or financial relationships that could be construed as a potential conflict of interest.
